# De Novo Structure Prediction from Tandem Mass Spectra: Algorithms, Benchmarks, and Limitations

**DOI:** 10.3390/molecules31050769

**Published:** 2026-02-25

**Authors:** Mark Yu. Schneider, Daniil D. Kholmanskikh, Kirill Ya. Romanov, Elena A. Perekina, Sergei A. Nikolenko, Ruslan Yu. Lukin, Ivan V. Golov

**Affiliations:** Research Center of the Artificial Intelligence Institute, Innopolis University, 420500 Innopolis, Russia

**Keywords:** de novo structure elucidation, mass spectrometry, machine learning, generative models, tandem mass spectrometry, cheminformatics, diffusion models, metabolomics

## Abstract

The identification of unknown molecules from analytical data remains a fundamental challenge in chemistry, with critical implications for drug discovery, metabolomics, and natural product research. While tandem mass spectrometry provides rich structural fingerprints, most spectra are absent from reference libraries, spurring the development of de novo generative models. However, their true accuracy has been difficult to assess. Our critical analysis reveals that state-of-the-art models achieve only 4.1% top-10 accuracy on rigorously leakage-controlled benchmarks like MassSpecGym. This sobering figure stands in stark contrast to earlier, overly optimistic reports, a discrepancy we attribute to pervasive data leakage in naive data splits. This review traces the field’s rapid evolution through three architectural eras: from fingerprint-conditioned RNN pipelines to end-to-end sequence models and, most recently, to graph-native diffusion under molecular-formula constraints. We demonstrate that explicitly conditioning generative models on a molecular formula significantly improves exact-match accuracy compared to unconstrained baselines. Crucially, our analysis distinguishes between two experimentally relevant paradigms: formula-conditioned generation for true unknown discovery and scaffold-based generation for hypothesis-driven research. While the latter shows high potential with oracle scaffolds, its performance drastically drops with predicted ones, revealing a critical bottleneck. To build the next generation of reliable tools, we propose a clear roadmap centered on standardized, leakage-aware benchmarking and transparent reporting.

## 1. Introduction

De novo structure elucidation from tandem mass spectrometry (MS/MS) remains a central challenge that limits our ability to chart the “dark” regions of chemical space [1] across drug discovery, metabolomics, and environmental analysis [2].

Specifically, this review addresses the question: how far can current machine learning models reliably predict the exact 2D molecular structure of a single, purified small molecule directly from high-resolution ESI-MS/MS data? In terms of scope, we focus exclusively on single-compound, high-resolution ESI-MS/MS and exclude mixture deconvolution, low-resolution ionization, and EI-MS, which provide fundamentally different information content. This deliberate focus on the “simplest-case” scenario is motivated by the fact that even this task remains far from solved. Reliably addressing it is a prerequisite for tackling more complex real-world challenges, such as different ionization methods or mixture analysis.

This task differs from adjacent components of the annotation pipeline: (i) formula identification—inferring elemental composition from MS1/MS2 and isotopic patterns [3]; (ii) fingerprint prediction and retrieval—projecting a spectrum into a molecular fingerprint for database ranking [4]; and (iii) candidate re-ranking—rescoring externally generated candidates [5]. By contrast, we examine (iv) genuine de novo generation that produces a molecular structure without relying on any candidate library.

Recent community estimates show that only 2–13% of spectra can be confidently matched, depending on the library and evaluation criteria [6], leaving most spectra unassigned. Although this number has improved from 1.8% in 2015 [1], the majority of chemical space remains unannotated. This persistent gap motivates learning-based generative approaches. On standardized HR ESI-MS/MS benchmarks such as MassSpecGym, a discrete graph-diffusion model conditioned on the true molecular formula achieves approximately 4.1% top-10 exact-match accuracy, outperforming scaffold- or sequence-based baselines [7]. These results indicate that explicit chemical constraints both shrink the generative search space and improve exact- and close-match recovery. We synthesize and critically compare recent architectures, datasets, and evaluation practices in de novo structure prediction from MS/MS, emphasizing how formula-aware graph diffusion reshapes achievable accuracy and evaluation realism.

Against this backdrop of rapid architectural progress, a critical re-examination of data practices and evaluation standards is essential to distinguish genuine advances from overfitting. In this review, we first introduce a unified taxonomy tracing the field’s evolution from decoupled RNN pipelines and end-to-end Transformers to the recent wave of graph-native diffusion models with explicit constraints. Second, we critically analyze available MS/MS data sources and highlight prevalent issues with data leakage, proposing a rigorous evaluation protocol to ensure reproducibility. Furthermore, we summarize essential chemistry-aware preprocessing and formula-conditioning practices required for practical implementation. Finally, we identify persistent bottlenecks—ranging from spectral representation to architectural trade-offs—that currently limit the robust deployment of de novo generation systems.

This review covers works published between 2020 and 2025, identified through targeted searches in PubMed, Google Scholar, and arXiv. Section 2 provides the necessary background on data representation and preprocessing. Section 3 presents a taxonomy of methods. Section 4 and Section 5 examine datasets and evaluation metrics, respectively. Section 6 discusses challenges and future directions.

## 2. Background

Reverse MS annotation critically depends on how spectra are preprocessed and how both spectra and molecules are represented for learning. These choices are affected by the heterogeneity of spectral databases and by the diversity of machine learning approaches proposed for MS/MS annotation. In practice, effective MS/MS–ML pipelines rely on two principles: (i) rigorous preprocessing that standardizes spectra and suppresses artifacts; (ii) chemically meaningful representations of both spectra and molecules.

### 2.1. Spectra Representation and Preprocessing

**Figure 1:**
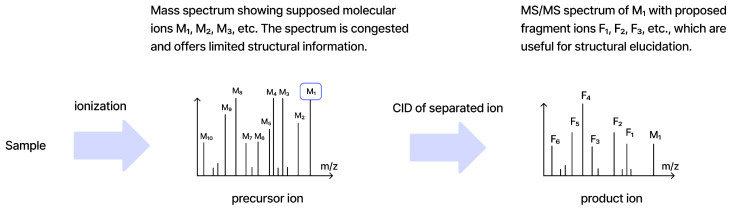
Tandem mass spectrometry flowchart.

An MS/MS spectrum is a set of peaks with mass-to-charge (m/z) and intensity (Figure 1). The information content depends on the resolving power of the instrument and on the fragmentation conditions. Below, we outline how spectra are encoded for learning [8].

High resolving power enables ppm- to sub-ppm-level mass accuracy [9] and reliable subformula assignment. Low resolution complicates formula searches and inflates candidate sets, motivating models that do not rely solely on exact formula prediction [10,11].

Electron Ionization (EI) and Electrospray Ionization (ESI) are the most common ionization methods for ML-oriented MS workflows. ESI typically preserves the precursor ion and adducts, whereas EI produces extensive fragmentation and may suppress the molecular ion. Tandem MS with collision-induced dissociation (CID) yields fragment spectra carrying structural information beyond precursor mass [12].

Acquisition parameters, most notably collision energy (CE) and stepped energies, shape fragmentation patterns. Some models merge spectra acquired at different CE values into consolidated MS/MS spectra, as in MSNovelist [13] and ICEBERG [14], while others encode CE explicitly as an input feature, as in MassFormer [15]. Spec2Mol demonstrated that combining low- and high-energy spectra can provide complementary information [16], although naive overlays may lose energy-dependent context [17]. Several recent models omit explicit CE conditioning and rely on data diversity, which simplifies deployment at the cost of reduced controllability [2,16,18,19,20].

**Figure 2:**
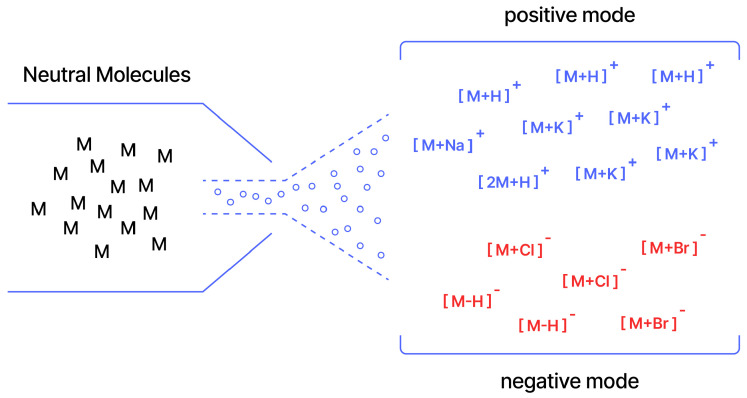
Ionization modes.

Ion mode and adduct type (Figure 2) also alter fragmentation pathways. Most datasets and models operate in positive mode and frequently restrict to ${\left[M+H\right]}^{+}$ [2,13,16,18,19,20,21], with occasional separate models for negative mode [22]. Broader adduct sets are sometimes included [22,23], but require explicit conditioning or augmentation. For example, DreaMS applies stochastic m/z shifts during training to reduce adduct anchoring [2].

Raw MS/MS data vary across matrices and instruments; therefore, preprocessing is mandatory before any learned representation. We consider five core steps: (i) noise suppression, (ii) mass-axis recalibration, (iii) intensity normalization, (iv) optional deisotoping, and (v) peak filtering (e.g., top-*N*). These steps standardize spectra across runs and reduce spurious variability prior to feature construction [24].

**Figure 3:**
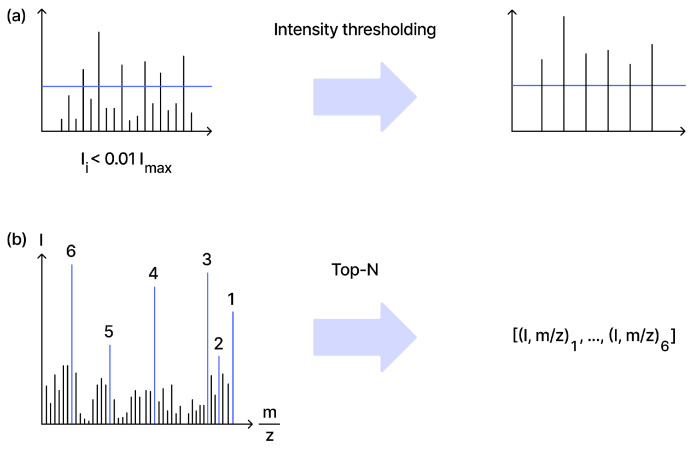
(**a**) Denoising via intensity thresholding; (**b**) denoising via top-*N* peak selection.

Noise and background removal target chemical and electronic artifacts. Common strategies include relative or absolute intensity thresholds [19,21,25] and peak-count caps (top-*N*) to control sparsity [16,18,23,24] (Figure 3). Wavelet-based and learned denoising approaches have also been explored [26,27].

Spectral recalibration aligns measured m/z values to trusted references, correcting systematic mass errors and improving fragment-matching. Without recalibration, mass errors for low-intensity peaks can reach tens of ppm. Recalibration can use internal standards, fixed-mass ions, or alignment to reference peaks with reliable formulas [28].

Intensity normalization reduces inter-run variability and enables cross-instrument comparisons [29]. Approaches include total ion current scaling [30], normalization to internal standards labeled with stable isotopes [31], square-root or logarithmic transforms that compress dynamic range and up-weight informative low peaks [21,32], physically motivated normalization schemes [33], scaling relative to the base peak [21,22,34,35], and min–max scaling [36].

(1) $$\begin{array}{cc} \mathrm{Relative}\;\mathrm{scaling}:\; & I_{i}^{\prime }=\frac{I_{i}}{\max(I)}, \end{array}$$

(2) $$\begin{array}{cc} \mathrm{Min}–\max\;\mathrm{normalization}:\; & I_{i}^{\prime }=\frac{I_{i}-\min\left(I\right)}{\max(I)-\min(I)}. \end{array}$$

Deisotoping removes isotope peaks that do not carry structural information for fragment ions. It is particularly useful when workflows infer or exploit molecular or subfragment formulas [3,30,33,37]. Accurate formulas can also aid evaluation [16], candidate filtering [32], and generation without surplus atoms [38]. Gradient-boosting models such as XGBoost and CatBoost have been applied to assign isotopic peaks in FT-ICR MS spectra [39], but require ultra-high-resolution data and synthetic training spectra. Transformer encoders trained directly on raw peaks can tolerate some isotopic redundancy [2]; thus, deisotoping remains optional and should match the downstream representation and compute budget.

**Figure 4:**
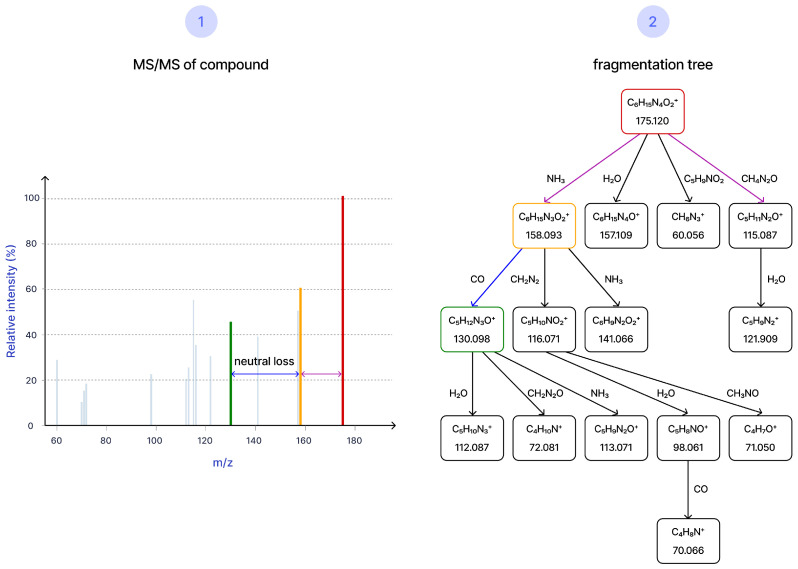
Fragmentation-tree representation of MS/MS.

After preprocessing, spectra are converted into model-ready representations. A widely used option, implemented for example in SIRIUS, is a graph-based fragmentation tree (Figure 4): nodes represent annotated ion fragments, and edges represent neutral losses connecting them in a fragmentation cascade [40]. This graph encodes chemically plausible fragmentation paths. Earlier tree-building algorithms relied on heuristic scoring that could fail for certain chemotypes [3], especially for high-mass analytes; recent work combining fragmentation trees with transformer models has improved robustness [41].

Binning is another common representation strategy. The m/z range is divided into fixed-width bins, and each bin stores an aggregated intensity (e.g., sum, mean, or maximum) [18,32,42]. Choosing bin widths comparable to instrumental resolution simplifies computations but discards fine isotopic patterns and can distort fragment intensities. Ignoring the dependence of resolution on m/z leads to peak aggregation at low masses and peak clipping at high masses. To mitigate this, adaptive binning schemes with m/z-dependent widths have been proposed [25].

Sequence-based representations treat a spectrum as an ordered list of the top-*N* peaks (m/z,I), usually retaining the most intense signals [2,23,25,34]. The precursor peak may be excluded, and random peak masking is often applied to regularize training and promote learning of fragmentation patterns [2,18,23]. Continuous m/z values can be discretized into tokens, while subformula tokens [7,41] provide chemically interpretable vocabularies at higher computational cost and increased sensitivity to calibration errors.

Several encoders map m/z tokens to smooth high-dimensional features using Fourier-based positional encodings [43]:

(3) $$\begin{array}{cc} \Phi (m/z,2i) & =\sin(2\pi \cdot m/z\cdot F_{i}), \end{array}$$

(4) $$\begin{array}{cc} \Phi (m/z,2i+1) & =\cos(2\pi \cdot m/z\cdot F_{i}). \end{array}$$

For an m/z value, the encoding uses a set of frequencies from $F_{i}$ elements that span low and high frequency bands. In DreaMS, this design yields encodings where small m/z shifts induce small feature changes, matching ppm-level tolerances [2]. Related ideas appear in Mass2SMILES [19] and IDSL_MINT [44].

Learned spectral embeddings are dense, low-dimensional vectors produced by neural encoders trained on large spectral collections. They are more efficient than sparse vectors for computationally intensive tasks and can capture holistic spectral patterns rather than individual peaks [17,45]. This often improves robustness to minor m/z shifts and experimental variation. Self-supervised objectives are particularly attractive under limited chemical coverage; DreaMS is one recent example that combines peak-level Fourier features with transformer encoders into a composite spectral embedding [2].

The PeakEncoder in DreaMS receives raw mass spectra as a matrix $M\in R^{2\times (n+1)}$,

(5) $$M=\left(\begin{array}{cccc} m_{0} & m_{1} & \ldots & m_{n}\\ 1.1 & I_{1} & \ldots & I_{n} \end{array}\right),$$

augmented with a special precursor token. Integer and fractional parts of m/z are mapped to sinusoidal components, which, together with raw intensities, are processed by feed-forward networks to form peak tokens. Because peaks form a set rather than an ordered sequence, no positional encoding is used.

The SpectrumEncoder then applies a stack of transformer encoder layers with multi-head self-attention to these peak tokens. A Graphormer-like [46] modification injects neutral-loss information via the same Fourier features, making the architecture permutation-equivariant with respect to peak order. The final hidden state of the precursor token serves as the aggregated DreaMS embedding for the spectrum.

### 2.2. Molecular Representation

Molecular representations determine both the feasibility of valid de novo generation and how effectively models can align spectral evidence with structural hypotheses. We review (i) string encodings, (ii) molecular fingerprints, (iii) graph-based 2D representations, (iv) 3D conformations, and (v) molecular embeddings used in subsequent sections.

String encodings such as SMILES are compact but syntactically fragile: small token edits can yield invalid or chemically inconsistent graphs [47,48,49]. Limitations include ambiguous implicit hydrogens, incomplete treatment of tautomers and stereochemistry, valence violations in charged species, and brittle ring-closure syntax [50,51]. SELFIES encodes valence constraints directly in the grammar, delivering near-100% syntactic validity and greatly reducing invalid outputs, while DeepSMILES modifies SMILES syntax to stabilize generation without enforcing full valence constraints [50,52].

Molecular fingerprints [53] map topology and functional groups into fixed-length vectors and support property prediction, retrieval, and evaluation (e.g., Tanimoto-based similarity in Section 5) [7,15,36,44,54]. Morgan fingerprints and their extended connectivity variants (ECFP4/ECFP6) are widely used; bit collisions can occur when different substructures map to the same bit [55,56], and increasing dimensionality (2048–4096 bits) or using collision-aware designs mitigates this. More specialized schemes further reduce collisions and improve retrieval robustness [57].

Graph representations align naturally with the de novo target: atoms are nodes, bonds are edges, with node features (element, formal charge, chirality, radical electrons) and edge features (bond order, aromaticity) [58]. Consistent handling of charges, aromaticity, and stereochemistry is crucial for valid generation and fair evaluation.

Three-dimensional representations include point clouds and geometric graphs, where each atom has coordinates and features [59,60]. These capture shape but require separate connectivity information and can hinder end-to-end differentiation for complex molecules [61]. Many recent models therefore treat 2D molecular graphs as the primary connectivity scaffold from which 3D conformations are derived [54,62,63,64].

For coordinate-level models, SE(3) invariance and equivariance are essential. Invariant descriptors remain unchanged under rigid motions; equivariant networks (e.g., EGNN, SchNet, PaiNN, G-SphereNet) transform consistently with rotations and translations and are now standard for 3D generative models [65,66,67].

Molecular embeddings are low-dimensional vectors summarizing topology and chemistry for tasks such as comparison and generation. Hybrid encoders that combine string- and graph-based inputs are increasingly common [68], but for de novo generation from MS/MS, graph-based embeddings are particularly well aligned with the graph-native generators discussed in Section 3.

## 3. Taxonomy of Methods

**Figure 5:**
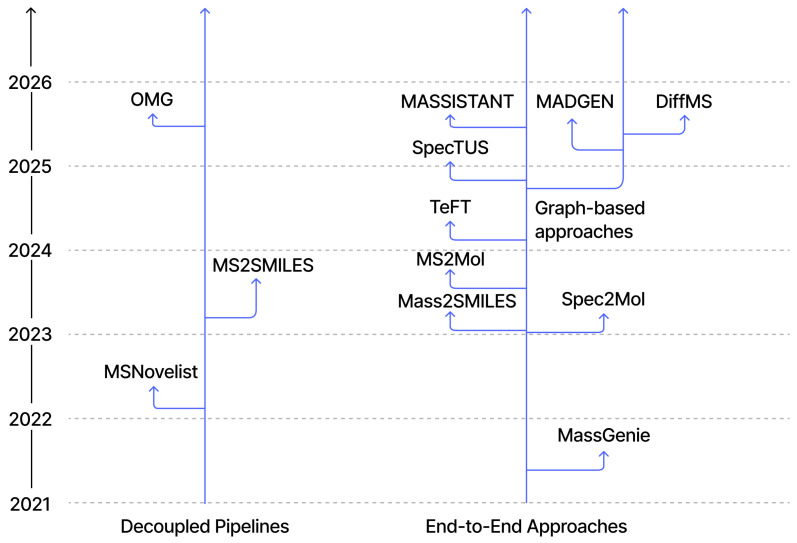
Architectural evolution of de novo structure generation models. Models are organized along a timeline and grouped into three paradigms: decoupled pipelines, end-to-end sequence models, and graph-based approaches.

The evolution of de novo structure generation from mass spectra follows a clear trajectory toward more direct, chemically constrained modeling [7,13,16,18,19,23,25,32,35,38,69]. Three major categories can be distinguished: (1) decoupled pipelines that mitigate data scarcity via intermediate fingerprints and external tools; (2) end-to-end sequence-to-sequence models that treat spectra as a language to be translated into SMILES/SELFIES; (3) graph-native and diffusion-based methods that generate molecular graphs directly. This trajectory is summarized in Figure 5.

### 3.1. The Unified Supervised Learning Framework

**Figure 6:**
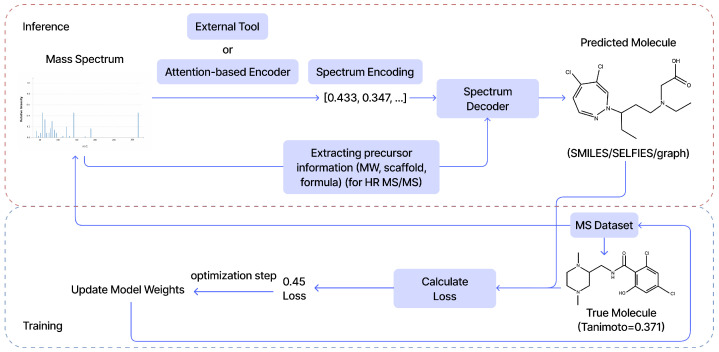
General supervised learning framework for de novo molecular generation from mass spectra. Inference: the spectrum is encoded and decoded into a candidate structure, optionally constrained by precursor information. Training: the model minimizes a loss derived from similarity between predicted and ground-truth structures.

Regardless of architecture, most modern approaches share a common supervised framework (Figure 6). During Inference, a spectrum is encoded into a numerical representation (utilizing Transformers, CNNs, MLPs, or external tools such as CSI:FingerID), which a decoder then maps to a molecular structure (SMILES, SELFIES, or graphs). Crucially, these models can be conditioned on auxiliary “oracle” information, such as molecular formulas or scaffolds, to prune the generative search space. During Training, the model is optimized by minimizing a loss function derived from the discrepancy between the generated structure and the ground truth, often mediated by the similarity metrics discussed in Section 5.

### 3.2. Decoupled Pipeline Approaches

The primary obstacle for deep-learning-based de novo generation is the scarcity of labeled high-resolution MS/MS spectra, which are available for orders of magnitude below the datasets typically required to train generative models from scratch. To address this data bottleneck, early methods adopted a modular, decoupled strategy: decomposing structure prediction into a spectral annotation step (predicting fingerprints or formulas by external tools) followed by a structural generation step.

MSNovelist [13] pioneered this approach by bridging the gap between spectral data and structural generation via intermediate molecular descriptors. It utilizes CSI:FingerID to predict structural fingerprints and SIRIUS to determine molecular formulas, feeding these as conditioning variables into an LSTM-based SMILES decoder trained on ∼1.2 M database structures. Building upon this modular logic, MS2SMILES [38] sought to improve the reliability of the generated structures by introducing explicit chemical constraints. By treating hydrogen atoms as implicitly coupled to heavy atoms and enforcing SMILES grammatical rules during decoding, MS2SMILES improved exact-match accuracy and reduced the production of chemically invalid strings.

Despite these advancements, these early decoupled architectures share two critical vulnerabilities. First, they are highly susceptible to error propagation [13]: since the structural generator is strictly conditioned on the predicted fingerprint, any inaccuracies in the initial spectral annotation establish an impassable performance ceiling for final structure recovery. Second, the seemingly high accuracies reported by these models (e.g., 45% top-*k* inclusion for MSNovelist and 45.1% top-10 for MS2SMILES) must be re-evaluated in the context of evaluation realism. Our critical analysis suggests these figures were likely inflated by pervasive data leakage in the original GNPS splits, where structurally similar molecules appeared in both training and test sets.

A distinct solution to the error propagation problem was proposed with OMG [69]. Rather than relying on fixed fingerprints, OMG utilizes transfer learning to fine-tune a pretrained REINVENT4 [70] generator on molecules from PubChem that match a specific target formula. Guided by reinforcement learning and ranking models like JESTR, OMG demonstrated that de novo models could effectively explore chemical space even when experimental training spectra are limited.

The decoupled paradigm, while enabling early progress despite limited training data, faced inherent limitations. Its primary bottleneck was error propagation from upstream predictions: inaccuracies in the initial spectral annotation (fingerprint or formula) imposed an impassable ceiling on final structure recovery. Moreover, because the generative model was trained independently of raw spectral features, it could not learn to correct or adapt to spectral ambiguities. These constraints ultimately motivated the shift toward end-to-end architectures discussed in the following section. Nevertheless, several foundational ideas from this era remain central to contemporary graph-based models: (i) explicit conditioning on molecular formulas to constrain the search space, now standard in graph-based diffusion models; (ii) the value of modular pretraining, realized today in separate spectral encoder and graph decoder pre-training; and (iii) leveraging large structure-only databases to compensate for scarce spectrum–structure pairs.

### 3.3. End-to-End Sequence Translation Models

Inspired by advances in NLP, subsequent models framed de novo generation as direct translation from spectra to molecular strings.

The first major attempt to implement this was MassGenie [18], which utilized a Transformer encoder–decoder architecture. By pretraining on millions of in silico spectrum-SMILES pairs, MassGenie demonstrated that Transformers could learn the latent “grammar” of molecular fragmentation. However, this unconstrained approach revealed a significant sim-to-real gap: models trained predominantly on synthetic data often struggle to generalize to the noise and complexity of experimental spectra.

To address the lack of chemical mass-fidelity in early translation models, MS2Mol [23] introduced high-resolution m/z tokenization and incorporated the precursor mass as a specific input feature. By using Byte Pair Encoding (BPE) for SMILES strings, MS2Mol improved the recovery of common chemical substructures. Crucially, it demonstrated that providing the true molecular formula as an “oracle” constraint during inference could lift close-match accuracy by several percentage points.

While sequence-to-sequence models reduced pipeline errors, they introduced a new vulnerability: syntactic fragility. Because SMILES strings are highly sensitive to single-token errors, models like Mass2SMILES [19] and Spec2Mol [16] experimented with multi-task learning and pre-learned chemical latent spaces to stabilize generation. MASSISTANT [35] took this a step further by adopting SELFIES representations, which ensure chemical validity by construction, thereby eliminating the production of non-physical molecular strings.

This era also saw the adaptation of translation models to specialized, “information-poor”regimes. SpecTUS [32] tackled the challenges of GC-EI-MS, where the frequent absence of the molecular ion makes structure recovery particularly difficult, by leveraging multiple synthetic data sources during pre-training. Similarly, TeFT [11] addressed the noise and accuracy limitations of low-resolution ESI-MS/MS from miniaturized spectrometers. By implementing a hybrid re-ranking strategy that validates neural proposals against in silico fragmentation trees, TeFT demonstrated that even lower-fidelity analytical data can yield viable structural hypotheses.

Despite these advancements, a fundamental limitation remains for all sequence-based models: they impose a linear sequence on molecular graphs that are inherently non-sequential. This artificial ordering limits the model’s ability to capture global structural symmetries and branching patterns, ultimately motivating the shift toward the graph-native and diffusion-based processes discussed in the next section. Nevertheless, sequence-based models contributed lasting innovations that inform current architectures: (i) the Transformer encoder, now integral to graph-based spectral encoders; (ii) the demonstration that oracle formula conditioning improves accuracy, directly motivating formula constraints in diffusion models; and (iii) the exploration of robust string representations, which highlighted the importance of syntactic validity—a challenge inherently addressed by graph-native methods that operate directly on molecular topology.

### 3.4. Graph-Based Diffusion Models

Recent state-of-the-art methods operate directly on molecular graphs, aligning model representations with chemical structure. Crucially, these graph-native architectures are the first to break the 0% exact-match accuracy barrier on the rigorously curated MassSpecGym benchmark, where previous sequence-to-sequence models frequently fail due to the benchmark’s strict leakage control and chemical complexity.

To integrate structural intuition into the generative process, MADGEN [25] introduced a two-stage scaffold-based pipeline. It first utilizes contrastive learning to retrieve a molecular scaffold (the “core backbone”) from a candidate pool, then employs an attention-based graph Transformer to “decorate” this scaffold via a Markov bridge process. While this method is highly interpretable and mirrors the hypothesis-driven workflow of natural product discovery, it has revealed a significant scaffold bottleneck. Our analysis of the MADGEN results shows a dramatic performance collapse: while the model achieves 12.4% top-10 accuracy when provided with an “oracle” (perfect) scaffold, its accuracy drops to just 1.54% when relying on its own predictive retriever. This discrepancy underscores that for scaffold-conditioned models, the retrieval of the correct chemical backbone remains a primary limiting factor.

In contrast, DiffMS [7] established a more resilient paradigm for the discovery of true unknowns by utilizing discrete graph diffusion. DiffMS sidesteps the scaffold retrieval problem by performing denoising directly on the molecular adjacency matrix, conditioned on a molecular formula. To overcome the scarcity of paired spectrum-structure data, DiffMS utilizes a dual-pre-training strategy: the spectral encoder is pre-trained to predict molecular fingerprints, ensuring it extracts physically meaningful features from raw peaks, while the diffusion decoder is pre-trained on a massive scale using 2.8 M fingerprint-structure pairs. By fixing the heavy-atom composition as a “hard constraint” and leveraging virtually infinite structure-only data during pre-training, DiffMS achieves 4.1% top-10 accuracy on MassSpecGym—more than double the accuracy of fully predictive scaffold-based pipelines. Nevertheless, it must be emphasized that while such accuracy represents the current state of the art, this absolute performance remains remarkably modest. This sobering figure underscores that de novo elucidation from a single spectrum remains one of the most formidable challenges in machine learning for chemistry. The fact that over 95% of compounds in leakage-controlled benchmarks still elude exact structural recovery highlights the inherent information scarcity of tandem MS and the need for the multimodal strategies discussed in the following section.

The emergence of graph-native models like MADGEN and DiffMS highlights a crucial dichotomy in problem formulation for de novo structure elucidation, centered on the type of available prior information: the molecular scaffold versus the molecular formula. These two scenarios are not merely different computational tasks; they represent distinct, and equally valid, experimental realities. Therefore, a direct comparison does not yield a single “best” model, but rather reveals the strengths of two specialized approaches. MADGEN demonstrates the immense potential of guided generation once a strong structural anchor is found, while DiffMS sets the state-of-the-art for generation from minimal chemical constraints. This underscores a key takeaway for the field: the development of both types of models is critical, and future benchmarks must clearly distinguish between these two evaluation scenarios to accurately reflect a tool’s practical utility for different chemical research questions.

As summarized in our comparison (Table 1), the consistent trajectory across all three eras reflects a migration from fragile sequence-based outputs toward robust, chemically constrained graph representations. Despite this progress, fundamental limits remain. Models often cannot distinguish structural and stereochemical isomers [7]; annotated high-resolution MS/MS datasets are still too small to support truly universal foundation models [2]; and cross-instrument generalization remains a critical bottleneck for the field, as all models are trained on only one domain.

## 4. Datasets and Benchmarks

### 4.1. Experimental Data Sources

Public and commercial MS/MS databases vary significantly in terms of chemical coverage, ionization modes, spectral resolution, and data accessibility [81]. Although targeted metabolome libraries for *E. coli* [82], yeast [83], plants [84,85], and humans [74] are integrated into broader resources; they remain limited in structural diversity. Finally, while ${\mathrm{MS}}^{n}$ spectra contain the richest structural information, they are relatively scarce; consequently, most computational methods rely primarily on MS/MS data.

Prominent resources such as mzCloud [86] and METLIN [87] host extensive curated collections, comprising approximately 1.7 million spectra (32 k molecules) and 3 million spectra (935 k molecules), respectively. However, these platforms often restrict bulk downloads, which limits their utility for training large-scale models. Standard database entries typically include the molecular formula, exact mass, InChIKey, and essential acquisition metadata.

Among commercial libraries, NIST23 [78] is a widely utilized resource. It comprises approximately 2.4 million MS/MS spectra representing roughly 52,000 molecules, with explicit categorization of high- and low-resolution data. Despite this volume, the library covers only a fraction of the chemical space defined by PubChem, and expanding high-quality coverage remains an ongoing priority.

The GNPS Spectral Library [71] functions as a central open-access repository. It consolidates spectra from multiple databases, including MassBank, MoNA, and specialized collections (e.g., ReSpect, HMDB), alongside community contributions, totaling approximately 2.6 million MS/MS spectra. The availability of preprocessed, downloadable data facilitates machine learning applications, while future growth depends on the integration of high-quality experimental measurements and curated external datasets.

MassSpecGym [80] constitutes an open-access, high-resolution MS/MS dataset tailored for benchmarking small-molecule models. It integrates spectra from MoNA, MassBank, and GNPS with approximately 10,000 internal measurements, yielding a curated collection of roughly 230,000 spectra spanning 29,000 unique molecules. A distinguishing feature of MassSpecGym is the provision of MCES-based data splits designed to rigorously control data leakage. Furthermore, the associated GeMS repository comprises approximately 700 million unannotated spectra stratified into quality tiers (A, B, C); specifically, the GeMS-A10 subset (≈24 million high-quality, deduplicated spectra) is optimized for large-scale pretraining. Distributed under the MIT license, the dataset permits both academic and commercial utilization. Valid benchmarking with this resource requires the strict exclusion of designated test molecules from training datasets.

NPLIB1 [14] constitutes an alternative benchmark dataset, originally developed for the CANOPUS framework and subsequently adopted as a standalone resource. It contains 8030 positive-mode MS/MS spectra representing 7131 molecules sourced from NIST2020, GNPS, and MoNA, curated to ensure diverse compound class coverage. NPLIB1 complements MassSpecGym and facilitates molecular property prediction tasks, as evidenced by its application in JESTR [5].

As detailed in Table 2, the available resources encompass both extensive spectral libraries utilized for training and specialized datasets developed for benchmarking. While repositories such as NIST23 and GNPS provide large volumes of spectral data, the number of unique molecular structures is comparatively lower, ranging from approximately 7000 to 52,000 across these subsets. This distinction suggests that while spectral redundancy is high, structural diversity remains a constraint for developing models capable of generalizing to novel chemical spaces.

### 4.2. Synthetic Data Generation

Given that experimental libraries capture only a subset of the relevant chemical space, synthetic data generation has emerged as a critical strategy for augmenting training datasets. The forward modeling task—predicting MS/MS spectra from molecular structures—has reached a higher degree of maturity compared to the inverse problem. Several state-of-the-art forward models have demonstrated substantial retrieval performance on the NIST20 benchmark, including FraGNNet [88] (30.3% Top-1 accuracy), ICEBERG [14] (27.9%), MassFormer [15] (20.4%), and NEIMS [89] (19.9%). In contrast, the inverse task remains challenging due to the inherent ambiguity of reconstructing molecular graphs from overlapping fragment patterns. For instance, the state-of-the-art inverse model DiffMS achieves Top-1 accuracies of 2.30% on MassSpecGym and 8.34% on NPLIB1, highlighting the difficulty of resolving exact structures and stereochemical isomers solely from spectral data.

These performance metrics highlight the potential of utilizing synthetic MS/MS data for model pretraining and augmentation. A prevalent methodological strategy involves an initial pretraining phase on large-scale synthetic datasets to expand chemical space coverage, followed by fine-tuning on experimental high-resolution spectra to mitigate the domain gap.

This paradigm is exemplified by SpecTUS, which generates approximately 19 million synthetic GC-EI-MS spectra using the NEIMS and RASSP frameworks [90], followed by fine-tuning on experimental NIST20 data. Alternatively, MassGenie [18] employs synthetic molecular fragments produced by FragGenie to train a Transformer model for spectrum-to-SMILES translation. A distinct approach is utilized in OMG (Optimized de novo Molecular Generation), which combines synthetic and predicted spectra via ESP models [91]. In this framework, the spectrum of a candidate molecule is approximated as a weighted sum of two learned predictors, enabling the optimization of the generator to align predicted spectra with the target.

A complementary research direction involves the application of reinforcement learning to synthetic or hybrid datasets. Within this framework, generative models propose candidate molecular structures, which are subsequently evaluated by generating synthetic spectra and calculating reward signals based on their similarity to reference data. This approach effectively incorporates spectral alignment as a direct optimization objective.

Collectively, these methodologies indicate that synthetic data generation serves as a primary mechanism to address the scarcity of annotated experimental spectra. By leveraging forward models for data augmentation and reinforcement learning feedback, these strategies facilitate model training on a scale unattainable with experimental libraries alone, subject to the fidelity of the underlying simulation algorithms.

### 4.3. Data Leakage and Splitting Strategies

The validation of machine learning models in molecular sciences is fundamentally challenged by the non-independent and identically distributed nature of chemical data. Standard random splitting frequently leads to information leakage, where structurally analogous molecules appear in both training and test sets, inflating performance metrics without reflecting true generalization capability. To address this, the field has evolved through three distinct methodological paradigms: deterministic structural grouping, metric-space clustering, and combinatorial optimization.

#### 4.3.1. Structural and Metric-Based Heuristics

Initial strategies to mitigate information leakage relied on deterministic rules derived from molecular topology. Scaffold splitting became the standard baseline, partitioning datasets based on Murcko scaffolds to ensure that core ring systems remain disjoint between folds. However, this approach is increasingly recognized as overly rigid due to its tendency toward over-stratification. As demonstrated by Landrum [92], Murcko decomposition often fragments chemically coherent series into disparate subgroups; empirical analysis of ChEMBL assays reveals a median scaffold-to-compound ratio of approximately 0.4. This high granularity implies that nearly half of the molecules in a typical dataset are assigned unique scaffolds, resulting in artificially pessimistic evaluation regimes that fail to reflect the continuity of chemical space. To introduce topological nuance, Murcko Histograms were proposed [80], clustering molecules based on the frequency and connectivity of ring systems rather than binary scaffold identity.

A parallel trajectory moved away from discrete topology toward continuous similarity spaces. Sphere Exclusion (or Butina clustering) represents a distance-based heuristic that groups molecules using Tanimoto similarity on fingerprints. Enforcing a minimum distance between clusters offers a more chemically intuitive split than scaffold-based methods. However, Sphere Exclusion is inherently greedy and order-dependent; the resulting partitions are sensitive to initialization and do not guarantee a globally optimal separation of chemical space.

#### 4.3.2. Optimization-Based Partitioning

To overcome the sub-optimality of greedy heuristics, recent approaches formulate data splitting as a global optimization problem. Spectral Split [93] addresses the distribution shift problem by minimizing the normalized cut of the molecular similarity graph. This maximizes the covariate shift between training and test distributions, creating a rigorous test of extrapolation capabilities.

However, maximizing shift is not equivalent to minimizing leakage. To explicitly target the latter, the LoHi splitter [94] frames partitioning as a Balanced Vertex Minimum *k*-Cut problem, solved via Integer Linear Programming (ILP). While LoHi provides provably optimal leakage minimization, its computational cost scales poorly ($O(2^{n})$), making it intractable for datasets exceeding 50,000 molecules. This scalability bottleneck was recently resolved by DataSAIL [95], which employs a two-stage strategy: molecules are first grouped into granular clusters, and ILP is subsequently applied to these clusters rather than individual instances. This hybrid approach retains the mathematical rigor of global optimization while scaling to millions of entries.

#### 4.3.3. Domain-Specific Adaptation

General-purpose cheminformatics splitting strategies may not fully capture the signal requirements of specialized modalities. In mass spectrometry, fragmentation patterns are governed by specific structural breaking points rather than global substructure presence. To address this distinction, MassSpecGym utilizes an MCES-based splitting strategy based on the Maximum Common Edge Subgraph distance. By quantifying structural edit distance, this method aligns data partitioning with the topological characteristics of the fragmentation process.

However, structural splitting primarily addresses chemical novelty and does not account for spectral variance arising from instrument physics and acquisition parameters. As noted by Nguyen et al. [96], spectra for identical molecules can vary significantly depending on the mass analyzer (e.g., Orbitrap vs. Q-TOF) and collision energy settings. Consequently, relying solely on structural splits may lead to performance estimates that do not generalize to different experimental setups. To evaluate robustness against these physical variations, three additional stratification strategies are proposed:

- Cross-Instrument Splitting: This strategy partitions data by analyzer type. For instance, models trained on High-Energy Collision Dissociation (HCD) data from Orbitrap instruments (reported in Normalized Collision Energy, NCE) are evaluated on Collision Induced Dissociation (CID) data from Q-TOF instruments (reported in eV). This approach assesses the model’s capacity to learn instrument-agnostic fragmentation rules rather than relying on device-specific spectral features [96].
- Collision Energy Extrapolation: Spectral composition is sensitive to collision energy. Standard benchmarks often utilize averaged spectra, which may obscure performance limitations at specific energy levels. A more rigorous evaluation involves holding out specific energy ranges (e.g., training on 20/40 eV and testing on 30 eV). Methodologies such as SpIfII demonstrate that interpolating between discrete energies requires modeling the dynamics of bond dissociation [97].
- Precursor Ion Stratification: At lower collision energies, spectra are frequently dominated by the precursor ion with limited fragmentation. Nguyen et al. observe that including such spectra in the test set can result in high cosine similarity metrics without reflecting the model’s ability to predict fragment peaks. Robust evaluation implies stratifying test sets by the degree of fragmentation to ensure models are tested on dissociation patterns of varying complexity.

In summary, the evolution of data splitting strategies reflects a transition from generic validation schemes toward protocols that rigorously control for information leakage. While combinatorial optimization methods, such as DataSAIL, offer theoretical guarantees for structural generalization, they do not account for the physicochemical heterogeneity inherent to mass spectrometry. Consequently, robust evaluation frameworks must integrate both rigorous topological separation and instrument-aware stratification to accurately estimate model performance across diverse experimental conditions.

## 5. Evaluation Metrics and Comparative Analysis

### 5.1. Chemical Formula Evaluation

Predicting the molecular formula constitutes the foundational step in de novo structure generation. In the context of high-resolution MS/MS, this task is significantly constrained by isotopic patterns and precise precursor mass measurements, often rendering the determination of molecular ion composition unambiguous. Predictive performance is evaluated using Top-*k* Chemical Formula Accuracy.

Formally, consider a dataset comprising *N* spectra. For each spectrum *j*, a model outputs a ranked list of *k* candidate formulas ${\hat{CF}}_{j,1},\ldots ,{\hat{CF}}_{j,k}$, with $CF_{j}^{\mathrm{GT}}$ denoting the ground-truth formula. The metric is defined as:

(6) $$\mathrm{Top}\text{-}k\;\mathrm{CF}\;\mathrm{Accuracy}=\frac{1}{N}\sum_{j=1}^{N}1\left(\exists \;i\leq k:{\hat{CF}}_{j,i}=CF_{j}^{\mathrm{GT}}\right),$$

where 1(·) is the indicator function.

A similar Top-*k* framework is applied to structure-level metrics discussed in subsequent sections. In such contexts, evaluation aggregates results over the *k* highest-ranking candidates (e.g., based on maximum similarity or minimum distance) prior to averaging across the dataset.

### 5.2. Molecular Structure Evaluation

#### 5.2.1. Validity, Novelty, Uniqueness, Internal Diversity

To assess the generative performance, the set of generated molecular structures, $Z_{\mathrm{gen}}$, is evaluated relative to the training set, $Z_{\mathrm{train}}$. In accordance with established evaluation protocols [98,99], the following metrics are utilized:

(7) $$\begin{array}{c} \mathrm{Validity}=\frac{N_{\mathrm{valid}}}{N_{\mathrm{gen}}}, \end{array}$$

(8) $$\begin{array}{c} \mathrm{Uniqueness}=\frac{N_{\mathrm{unique}}}{N_{\mathrm{valid}}}, \end{array}$$

(9) $$\begin{array}{c} \mathrm{Novelty}=\frac{N_{\mathrm{novel}}}{N_{\mathrm{valid}}}, \end{array}$$

(10) $$\begin{array}{c} \mathrm{IntDiv}=1-\frac{1}{|Z_{\mathrm{valid}}\left|(\right|Z_{\mathrm{valid}}\left|-1\right)}\sum_{\begin{array}{c} Z_{i},Z_{j}\in Z_{\mathrm{valid}}\\ i\neq j \end{array}}\mathrm{sim}\left(Z_{i},Z_{j}\right), \end{array}$$

where $N_{\mathrm{gen}}$ denotes the total number of generated samples, while $N_{\mathrm{valid}}$ corresponds to the subset passing cheminformatics validity checks (e.g., valence rules verified by RDKit). $N_{\mathrm{unique}}$ represents the count of distinct valid structures, and $N_{\mathrm{novel}}$ quantifies valid molecules that are not isomorphic to any structure within $Z_{\mathrm{train}}$. The similarity function sim(·,·) is typically defined as the Tanimoto coefficient calculated over molecular fingerprints. Hereinafter, *Z* denotes a molecule when unambiguous.

#### 5.2.2. Top-k SMILES Accuracy

Following the determination of a molecular formula (whether predicted or provided by an oracle), the capability of a model to recover the molecular structure is assessed using Top-*k* SMILES Accuracy. In accordance with the limitations of mono-dimensional MS/MS data, which typically lack the resolution required to resolve chiral centers or double-bond geometries, we define this metric based on graph-level equivalence. Specifically, the evaluation is performed by comparing canonical SMILES strings with all stereochemical specifications removed. This ensures that the metric quantifies the model’s ability to reconstruct the correct chemical connectivity (molecular graph) rather than penalizing it for stereochemical nuances that are not reliably encoded in the fragmentation spectra.

This metric evaluates whether the ground-truth stereoisomer is present among the highest-ranked candidates. Formally, for a given spectrum *j*, let ${\hat{S}}_{j,1},\ldots ,{\hat{S}}_{j,k}$ denote the generated list of *k* candidate molecules represented as canonical SMILES strings, and let $S_{j}^{\mathrm{GT}}$ denote the canonical SMILES of the ground-truth structure. The metric is defined as:

(11) $$\mathrm{Top}\text{-}k\;\mathrm{SMILES}\;\mathrm{Accuracy}=\frac{1}{N}\sum_{j=1}^{N}1\left(\exists \;i\leq k:{\hat{S}}_{j,i}=S_{j}^{\mathrm{GT}}\right).$$

### 5.3. Tanimoto Similarity and Top-k Maximum Tanimoto Similarity

Given that exact structural recovery is challenging, evaluating the degree of structural resemblance is essential. The standard metric for this purpose is the Tanimoto similarity (or Jaccard index) calculated over binary molecular fingerprints $f(\hat{Z})$ and $f(Z^{\mathrm{GT}})$. It is defined as:

(12) $$S_{T}\left(\hat{Z},Z^{\mathrm{GT}}\right)=\frac{c}{a+b-c},$$

where *a* and *b* represent the count of active bits in $f(\hat{Z})$ and $f(Z^{\mathrm{GT}})$, respectively, and *c* denotes the number of shared active bits (intersection). The coefficient $S_{T}\in \left[0,1\right]$, where a value of 1 indicates identical fingerprint representations, and values approaching 1 signify high structural similarity. Although alternative coefficients (e.g., Dice, Cosine, Simpson, Sörgel) exist, comparative analyses have demonstrated that they typically correlate strongly with $S_{T}$, supporting its status as a robust default for small-molecule comparisons [100].

To evaluate model performance across a ranked list of predictions, the Top-*k* Maximum Tanimoto Similarity is defined. For each spectrum *j* with a set of candidate structures ${\hat{Z}}_{j,1},\ldots ,{\hat{Z}}_{j,k}$:

(13) $$\mathrm{Top}\text{-}k\;\mathrm{Max}\;\mathrm{Tanimoto}=\frac{1}{N}\sum_{j=1}^{N}\max_{i\leq k}S_{T}\left({\hat{Z}}_{j,i},Z_{j}^{\mathrm{GT}}\right).$$

### 5.4. Fraggle Similarity and Top-k Maximum Fraggle Similarity

Fraggle Similarity (FraggleSim) represents a fragment-based metric designed to prioritize the overlap of chemically meaningful substructures. Unlike exact fingerprint matches, it exhibits reduced sensitivity to minor local structural modifications compared to $S_{T}$ [101]. As with Tanimoto, values approaching 1 indicate a high degree of structural resemblance. Let $S_{F}\left(\hat{Z},Z^{\mathrm{GT}}\right)\in \left[0,1\right]$ denote the Fraggle similarity score. The metric is defined as:

(14) $$\mathrm{Top}\text{-}k\;\mathrm{Max}\;\mathrm{Fraggle}=\frac{1}{N}\sum_{j=1}^{N}\max_{i\leq k}S_{F}\left({\hat{Z}}_{j,i},Z_{j}^{\mathrm{GT}}\right).$$

### 5.5. MCES Distance and Top-k Minimum MCES Distance

The Maximum Common Edge Subgraph (MCES) distance provides a metric for quantifying dissimilarity directly based on molecular graph topology, offering a complementary perspective to fingerprint-based similarity. For two molecular graphs $\hat{G}=\left(\hat{V},\hat{E}\right)$ and $G^{\mathrm{GT}}=\left(V^{\mathrm{GT}},E^{\mathrm{GT}}\right)$, let $G_{c}=\left(V_{c},E_{c}\right)$ denote their maximum common edge subgraph. The MCES distance is defined as:

(15) $$d_{\mathrm{MCES}}\left(\hat{Z},Z^{\mathrm{GT}}\right)=\;|\hat{E}\left|+\right|E^{\mathrm{GT}}\left|-2\right|E_{c}|.$$

In this framework, lower values correspond to higher structural similarity, with a distance of 0 indicating graph isomorphism.

To aggregate performance over ranked predictions, the Top-*k* Minimum MCES Distance is defined as:

(16) $$\mathrm{Top}\text{-}k\;\mathrm{Min}\;\mathrm{MCES}=\frac{1}{N}\sum_{j=1}^{N}\min_{i\leq k}d_{\mathrm{MCES}}\left({\hat{Z}}_{j,i},Z_{j}^{\mathrm{GT}}\right).$$

### 5.6. Limitations of Structural Similarity Metrics

**Figure 7:**
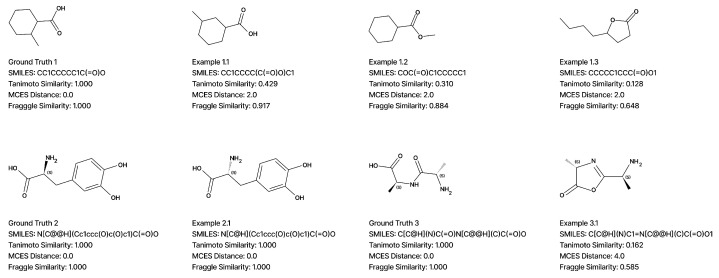
Illustrative values of structure similarity metrics for selected example molecules.

It is essential to emphasize that while fingerprint-based and fragment-based similarity metrics are standard for quantifying structural resemblance, they possess inherent limitations in generative settings, as illustrated in Figure 7.

First, metrics such as the MCES distance are subject to degeneracy. For instance, candidates from Example 1.1 through Example 1.3 all yield an identical distance despite representing structurally diverse modifications, ranging from positional isomerism to entirely different scaffolds, such as lactones. High similarity scores can frequently obscure incorrect functional group placements that may be biologically significant. Consequently, these values should not be interpreted as definitive indicators of near-correct structures, as even minor connectivity errors may involve structural isomers that participate in distinct metabolic pathways.

Second, Tanimoto similarity, Fraggle similarity, and MCES distance are fundamentally insensitive to stereochemical configurations. As demonstrated by the L-DOPA and D-DOPA pair (Ground Truth 2 and Example 2.1), identical scores are assigned to enantiomers despite their different biological roles. Since these metrics serve as the evaluation standard in the field, reported accuracy benchmarks typically reflect graph-level equivalence while omitting stereochemical information. Although the resolution of stereoisomers remains a critical objective, the application of fully 3D-aware metrics is currently constrained by computational scalability and the limited stereochemical resolution of standard MS/MS data.

Third, these metrics do not account for the complexities of gas-phase ion chemistry. The comparison between the linear dipeptide Ala-Ala and its cyclic oxazolone-type $b_{2}$-ion (Ground Truth 3 and Example 3.1) highlights a fundamental discrepancy in current evaluation strategies. While the $b_{2}$-ion is a direct mechanistic product of the precursor’s fragmentation, the metrics reflect a substantial structural mismatch: Tanimoto similarity is significantly diminished, the MCES distance increases, and fragment-based indicators such as Fraggle show a marked decrease. This occurs because standard algorithms are constrained by the connectivity of the static 2D graph and do not recognize the topological continuity between a linear precursor and its rearranged cyclic counterpart.

In experimental practice, phenomena such as adduct formation, in-source fragmentation, and complex gas-phase rearrangements occur extensively. These include hydrogen scrambling and proton migration [102,103], gas-phase cyclization of linear precursors [104], and rearrangements mediated by ion-neutral complexes [105]. Furthermore, skeletal and functional group migrations [106], charge-remote fragmentation [107], and even-electron variants of the McLafferty rearrangement [108] or retro-Diels-Alder reactions [109] produce fragment ions that structurally diverge from simple bond cleavages. Crucially, the majority of current de novo generators operate on static molecular graphs and do not account for these underlying chemical mechanisms. Consequently, standard metrics may inadvertently reward candidates that match artifact peaks or penalize correct structures that have undergone expected rearrangements. Therefore, these indicators should be viewed as measures of structural class resemblance rather than absolute proxies for definitive metabolite identification.

### 5.7. Recommended Evaluation Protocol

To establish a consistent and comparable benchmarking framework for de novo generation from MS/MS data, the following evaluation protocol is proposed: (i) Top-*k* Chemical Formula Accuracy; (ii) Top-*k* SMILES Accuracy (exact structure recovery); (iii) Top-*k* Max Tanimoto (primary metric for partial structural similarity); and, where computationally feasible, (iv) Top-*k* Max Fraggle and Top-*k* Min MCES.

Performance should typically be reported for ranks k∈{1,5,10}. When complemented by generative distribution metrics (Validity, Uniqueness, Novelty, and Internal Diversity), this suite of indicators facilitates robust cross-method analysis.

## 6. Challenges and Future Directions

This review has traced the evolution of de novo molecular structure prediction across three architectural paradigms, alongside the critical development of large-scale datasets and benchmarks. While graph-native diffusion models under explicit formula constraints currently define the empirical state of the art, our analysis reveals that the field is bounded by several critical methodological and physical bottlenecks. To build the next generation of reliable tools, the community must address the discrepancy between reported performance and experimental realism.

### 6.1. Key Empirical Insights and the Performance Gap

The current state-of-the-art is defined by two distinct and experimentally relevant paradigms: (a) formula-conditioned graph diffusion (e.g., DiffMS), which achieves ∼4.1% top-10 accuracy in a fully predictive setting suitable for unknown discovery, and (b) scaffold-conditioned generation (e.g., MADGEN), which reaches 12.4% top-10 accuracy when the correct scaffold is known. This distinction highlights the high potential of scaffold-based methods for hypothesis-driven research, such as library expansion or metabolomics of known pathways. However, a critical re-examination of the literature reveals that the seemingly high accuracy of early models (e.g., ∼45% top-*k* for MSNovelist) can be largely attributed to their evaluation on benchmarks with significant data leakage, such as the original GNPS splits. In contrast, the ∼4.1% top-10 accuracy of state-of-the-art models on the rigorously split MassSpecGym benchmark provides a much more realistic, albeit sobering, picture of current capabilities.

### 6.2. Methodological Standardizations and Evaluation Realism

The lack of standardized preprocessing and the presence of structurally biased dataset splits have historically led to metric inflation and misleading cross-paper comparisons. Inconsistent choices of fingerprints, similarity thresholds, and top-*k* definitions can often hide weak exact-match performance behind more permissive similarity scores. Moreover, the performance of de novo models is critically dependent on the type of prior information provided. As we have shown, the impact of an “oracle” scaffold is far more dramatic than an “oracle” formula; this makes direct cross-paradigm comparisons meaningless and underscores the necessity of evaluating models within their intended, realistic application scenarios. To ensure robust and comparable conclusions, the community must move toward standardized preprocessing and leakage-aware dataset splits (e.g., MCES-based). Based on our survey of the literature, we propose the following Standardized Evaluation Protocol:

- Minimum preprocessing protocol for HR ESI-MS/MS benchmarks: (i) consistent filtering by ionization mode and adducts; (ii) basic noise removal and top-*N* peak selection; (iii) m/z recalibration using internal or reference standards; (iv) intensity normalization (e.g., base-peak or TIC scaling); (v) optional deisotoping when using formula-aware models.
- Leakage-aware splitting: use MCES- or scaffold-based splits, as exemplified by MassSpecGym, instead of random molecule-level or spectrum-level splits.
- Core metrics: report at least Top-*k* SMILES exact-match accuracy and Top-*k* maximum Tanimoto similarity, with k=1,5,10 and clearly stated fingerprints. Complement these with topology-aware metrics such as Fraggle similarity and MCES distance, where relevant.
- Transparent conditions: always specify whether oracle or predicted formulas are used, which tool produced auxiliary predictions, and under which acquisition conditions (instrument, adducts, CE) results were obtained.

### 6.3. Data Scarcity, Domain Shift, and Self-Supervision

A major structural issue limiting de novo predictors is the scarcity of labeled high-resolution MS/MS data, which has historically forced models to rely on narrow experimental subsets. This results in poor generalization outside the training regime and leaves a significant gap between high-resolution ESI-MS/MS and other modalities like low-resolution or EI-MS data. To break this bottleneck, DreaMS [2] treats mass spectra as a language to be learned from the vast “dark matter” of metabolomics. Trained on the GeMS dataset comprising ∼201 million unannotated experimental spectra, DreaMS utilizes a Transformer architecture with two objectives: masked peak prediction to learn fragmentation dependencies (e.g., neutral losses), and retention order prediction to impart chromatographic logic. Crucially, the authors demonstrate via linear probing that structural awareness—specifically the ability to recognize molecular substructures (MACCS keys)—is an emergent property of this training. This suggests that future de novo generators could be initialized with such “foundation models” to bypass labeled data limitations, effectively separating the learning of spectral syntax from the learning of structural semantics.

### 6.4. Inherent Limitations and Multimodal Frontiers

While self-supervised foundations extract the maximum signal from spectra, they cannot transcend the fundamental physical limit of the technique: the inability to distinguish isomers with nearly identical fragmentation pathways. This necessitates a shift toward multimodal generative modeling, where generation is conditioned on orthogonal analytical signals.

Multimodal integration in structure elucidation is generally achieved through either early token-level serialization or modular representation-level fusion. The former strategy converts discrete spectral peaks and experimental metadata into tagged sequences or natural language prompts, allowing a single transformer to learn cross-modal correlations through self-attention within a unified sequence context [110,111]. Alternatively, specialized modality-specific encoders can extract distinct features that are subsequently merged via cross-modal attention layers to provide a holistic conditioning signal [112,113,114]. To effectively align these disparate signals, training protocols often utilize contrastive learning to map spectral and structural representations into a shared latent space, ensuring the model recognizes that distinct spectroscopic signatures belong to the same chemical entity [113,114]. This alignment is frequently reinforced by multi-task objectives—such as the concurrent prediction of functional group counts from fused embeddings—to ensure that integrated features prioritize chemically meaningful substructures [111,113]. Furthermore, modality dropout—randomly masking entire spectra during training—ensures the fusion mechanism learns to dynamically integrate any available subset of modalities rather than requiring all inputs simultaneously [110,111,112,113,114].

To illustrate the power of this paradigm, consider the classic challenge of distinguishing positional isomers, such as ortho-, meta-, and para- dihydroxybenzene. These compounds yield nearly indistinguishable mass spectra, making them intractable for MS-only models. Orthogonal modalities resolve this ambiguity through distinct physical principles:

1. Spectroscopic Validation via IR: IR spectra capture 3D-dependent features; for instance, the ortho- isomer forms an intramolecular hydrogen bond, resulting in a distinct O-H stretching profile distinct from the bands of meta- and para- isomers. A multimodal model can leverage this to assign high probability to the correct structure.
2. Chromatographic Filtering via Retention Time: Extending this logic to LC-MS, isomers exhibit different polarities and retention times (RTs). A generator conditioned on experimental RT could effectively prune candidates whose elution time significantly deviates from the measurement.

By conditioning generation on such combined evidence, models can transition from pattern matching to the holistic reasoning of an analytical chemist.

### 6.5. Practical Case Studies

To connect these architectural considerations to realistic laboratory workflows, we briefly outline two prototypical application scenarios in which de novo generators are already beginning to play a role.

First, in untargeted metabolomics, GNPS-style molecular networking frameworks [71] combine MS/MS spectra, spectral-library searches, and in silico MS/MS prediction [81] to organize unknown features into chemically coherent clusters. De novo structure generators such as MSNovelist, MS2SMILES, and MS2Mol [13,23,38] can be integrated at the level of each molecular feature: given a precursor ion, its MS/MS spectrum, and (when available) a molecular formula, the generator produces a ranked shortlist of candidate structures. These candidates are then filtered using orthogonal evidence routinely available in metabolomics workflows—consistency of adducts and neutral losses, retention time trends within a series, and biological plausibility with respect to pathway-focused metabolome databases such as ECMDB and YMDB [82,83]. In this setting, de novo models do not replace classical identification pipelines; rather, they act as hypothesis generators that accelerate the prioritization of plausible structures for subsequent targeted validation.

Second, in early drug discovery and ADME studies, de novo generators can assist in rationalizing unexpected MS/MS signals corresponding to unknown metabolites, degradation products, or process-related impurities. Starting from a parent drug scaffold and its known biotransformation chemistry, the MS/MS spectrum of a putative metabolite can be used to condition a generative model that proposes structures consistent with both the fragmentation pattern and plausible enzymatic or environmental transformations. The resulting candidates are cross-checked against curated metabolism databases [82,83] and, where applicable, multi-modal measurements such as LC retention times or orthogonal spectroscopic data. In this context, generative models serve as a structured way to enumerate and rank chemically reasonable hypotheses, guiding follow-up experiments rather than providing stand-alone, definitive structure calls.

### 6.6. Uncertainty Calibration

Finally, for deployment in real laboratories, models must provide calibrated confidence estimates and clear failure modes. Practical systems should expose probability-calibrated scores, abstain from low-confidence cases, and flag out-of-distribution spectra rather than returning overconfident structures.

### 6.7. Key Takeaways

In summary, de novo elucidation currently leads the field through two complementary paradigms: formula-conditioned and scaffold-conditioned generation. Neither is universally superior; their effectiveness is dictated by the specific chemical question and available experimental information. Robust future progress will require the adoption of standardized preprocessing, leakage-aware dataset splits, and the integration of multimodal constraints and calibrated uncertainty estimation. These elements are the essential ingredients for the next generation of practically useful de novo discovery tools.

Our analysis further confirms that providing the correct molecular formula consistently improves close-match and exact-match accuracy. Gains of several percentage points reported for methods such as MS2Mol and DiffMS demonstrate that formula constraints shrink the search space in a practically meaningful way. However, the impact of using a predicted versus an oracle molecular formula is highly architecture-dependent. For instance, scaffold-based models like MADGEN show a dramatic performance drop on MassSpecGym, where top-10 accuracy falls from 12.4% with oracle scaffolds to just 1.54% with predicted ones, revealing a severe backbone-retrieval bottleneck. Conversely, the end-to-end diffusion model DiffMS is remarkably resilient; its top-10 accuracy only decreases from 4.25% to 4.1% when using a predicted formula from MIST-CF. This suggests that for top-tier systems, formula prediction is becoming a less critical error source, shifting the core challenge back to resolving isomeric ambiguity from a given formula.

Despite these advancements, sequence models trained predominantly on synthetic spectra continue to exhibit a persistent sim-to-real gap, underperforming on noisy, heterogeneous experimental spectra if not carefully fine-tuned on curated HR data. Furthermore, all methods remain sensitive to choices of ion mode, adducts, collision energy, and instrument type. Consequently, reported state-of-the-art numbers should be interpreted as tied to specific acquisition regimes rather than being universally applicable.

## 7. Conclusions

This review has provided a comprehensive analysis of the rapid evolution of de novo molecular structure prediction from tandem mass spectra. By tracing the field’s trajectory across three distinct architectural eras—from decoupled RNN pipelines and end-to-end Transformers to graph-native diffusion models—we have characterized the transition from fragile string-based translation to chemically grounded topological generation. Our analysis reveals that while the field has made significant algorithmic strides, it is currently undergoing a necessary structural correction driven by the emergence of high-resolution, leakage-controlled benchmarks.

The most critical finding of this survey is the profound discrepancy between reported model performance and experimental reality. While early models on naive data splits suggested accuracies as high as 45%, rigorous evaluation on benchmarks like MassSpecGym reveals that current state-of-the-art models achieve closer to 4.1% top-10 accuracy. This “sobering” reality check underscores the pervasive issue of data leakage and emphasizes that structural similarity between training and test sets has historically led to over-optimistic success metrics. We conclude that MCES-based or scaffold-disjoint splitting must become the mandatory standard for all future de novo structure elucidation research.

Despite these challenges, the emergence of formula-conditioned graph diffusion represents a resilient frontier for true unknown discovery. However, algorithmic improvements alone cannot transcend the physical information limits of tandem mass spectrometry. To illuminate the “dark matter” of chemical space, the field must transition toward multimodal foundation models. We identify large-scale self-supervised pre-training (e.g., DreaMS) and the integration of orthogonal analytical modalities—such as IR spectroscopy and retention time—as the essential pathways to resolving structural isomeric ambiguity.

In summary, the next generation of de novo discovery tools must be built on the pillars of standardized preprocessing, leakage-aware evaluation, and multimodal integration. By addressing the current performance bottlenecks and moving toward calibrated, uncertainty-aware systems, these computational frameworks will eventually provide the robust structural hypotheses required to accelerate discovery in metabolomics, drug research, and environmental chemistry.

## Figures and Tables

**Table 1:** Comparative overview of representative de novo molecular generation models from mass spectra.

Model	Year	Output	Architecture	Conditioning	Benchmark
MassGenie [18]	2021	SMILES	Transformer	None	GNPS [71], CASMI [72] 2017
MSNovelist [13]	2022	SMILES	CSI:FingerID + LSTM	Formula	GNPS, CASMI 2016
MS2SMILES [38]	2023	SMILES	LSTM	Formula	GNPS, CASMI 2016
Spec2Mol [16]	2023	SMILES	CNN encoder + GRU decoder	MW (re-ranking)	NIST20 [73]
MS2Mol [23]	2023	SMILES	Transformer (BART-style)	Optional formula	CASMI 2022
Mass2SMILES [19]	2023	SMILES	Transformer + TCN	None	GNPS, NIST20, CASMI 2022
TeFT [11]	2024	SMILES	Transformer + fragment-tree re-ranking	Fragment tree (re-ranking)	GNPS, HMDB [74] 5.0, MoNA [75], CASMI 2017
SpecTUS [32]	2025	SMILES	Transformer (BART)	None (EI-MS)	NIST20, SWGDRUG [76], Cayman [77], MoNA
MASSISTANT [35]	2025	SELFIES	MLP	None (EI-MS)	NIST08
MADGEN [25]	2025	Graph	Contrastive encoder + GNN + graph Transformer	Scaffold, formula	NIST23 [78], CANOPUS [79], MassSpecGym [80]
DiffMS [7]	2025	Graph	Transformer + graph diffusion	Formula	CANOPUS, MassSpecGym
OMG [69]	2025	SMILES	RNN	Formula	CANOPUS, MassSpecGym

**Table 2:** Representative resources for MS/MS spectra used in de novo structure generation and evaluation.

Name	Purpose	MS/MS Spectra	Molecules	Type
NIST23	Data library	∼2.4 M	∼52 k	Commercial
GNPS Spectral Library	Data library	∼2.6 M	∼50 k	Free
MassSpecGym	Data library/Benchmark	∼230 k	∼29 k	Free (MIT)
NPLIB1	Benchmark	∼8 k	∼7 k	Free

## Data Availability

No primary research results, software or code have been developed as part of this review.
